# Anesthetic challenges of patients with cardiac comorbidities undergoing major urologic surgery

**DOI:** 10.1186/1755-7682-7-17

**Published:** 2014-04-29

**Authors:** Rudin Domi, Hektor Sula, Ilir Ohri, Arben Beqiri, Myzafer Kaci, Artan Bodeci, Haki Laho

**Affiliations:** 1 Department of Anesthesiology and Intensive Care, “Mother Teresa” University Hospital Center, Tirana, Albania; 2 Department of General Surgery, “Mother Teresa” University Hospital Center, Tirana, Albania; 3 Department of Medicine, Albert Einstein College of Medicine, Bronx, NY, USA

**Keywords:** Cardiac patient, Urological surgery, Anesthesia

## Abstract

The cardiac patient undergoing major urologic surgery is a complex case requiring a great attention by the anesthesiologist. Number of this group of patients having to go through this procedure is constantly increasing, due to prolonged life, increased agressiveness of surgery and increased anesthesia’s safety. The anesthesiologist usually has to deal with several problems of the patient, such as hypertension, chronic heart failure, coronary artery disease, rhythm disturbances, intraoperative hemodymanic changes, intraoperative bleeding, perioperative fluid imbalance, and metabolic disturbances. A cardiac patient undergoing major urologic surgery is a complex case requiring a great attention by the anesthesiologist. The scope of this review article is to present the most frequent issues encountered with this group of patients, and to synthetically discuss the respective strategies and maneuvers during perioperative period, which is the major challenge for the anesthesiologist.

## The predictors of perioperative cardiac events and risk stratification

Cardiovascular diseases remain the first worldwide cause of mortality [1]. Cardiac complications present significant risks to patients undergoing major noncardiac surgery. Perioperative risk stratification helps the anesthesiologist with appropriate treatment in patients who are at increased risk for such procedures. There are several controversies and debates regarding the risk factors of coronary heart disease and their direct contribute in perioperative cardiac events [2].

The risks of perioperative cardiac complications include evaluation of the presence or absence of clinical predictors of increased perioperative cardiovascular risk, cardiac functional status, and the surgery - related risks. Perioperative cardiac events are severe complications after noncardiac surgery in cardiac patients [3-5]. The incidence of perioperative cardiac events is approximately 1% for a general surgery population [6]. According to Lee [7], six independent predictors of complications were identified and included in a well-known Revised Cardiac Risk Index. These variables are: high-risk type of surgery, history of ischemic heart disease, history of congestive heart failure, history of cerebrovascular disease, preoperative treatment with insulin, and preoperative serum creatinine 2.0 mg/dl. Lee also reported the incidence of perioperative cardiac events varied from 0.5% (if no factors present) to 9% if more than 3 factors are present. Therefore, the Revised Cardiac Risk Index is a suitable tool to identify patients at risk to have perioperative cardiac events. Several authors [8] believe that Lee’s Revised Cardiac Risk Index must include patient age and emergency medicine as well. According to Boersma’s [8] study, 1.7% of the enrolled patients died perioperatively. Fleisher [9] and Eagle summarized six predictive factors of perioperative increased cardiac risk including ischemic heart disease, heart failure, high risk surgery (intraperitoneal, intrathoracic, vascular), diabetes mellitus requiring insulin, renal failure, and poor functional capacity.

The preoperative optimization techniques such as coronary revascularization and β-blockade demonstrated controversial benefits excepting the highest-risk patients [10,11].

It is usually accepted that major intraoperative hemodynamic changes, such as hypotension and tachycardia, may induce perioperative cardiac events. Kheterpal et al. found that mean arterial pressure (MAP) ≤ 50 mmHg, or a 40% reduction from preoperative baseline MAP, is associated with perioperative cardiac complications. They also showed that tachycardia > 100 beats/min may increases the likelihood of cardiac events. The American College of Surgeons–National Surgical Quality Improvement Program (ACS-NSQIP) tended to find a correlation between hemodynamic changes and perioperative cardiac events in a 30-day follow up [12].

Recently, it has been shown that respiratory rate, heart rate, systolic blood pressure, and the level of consciousness are important predictors of mortality in hospitalized patients [13,14].

The genetic background of cardiovascular diseases must be considered. Several interesting studies [15] found that calcium/calmodulin-dependent kinase IV (CaMKIV) has an important role in blood pressure regulation. These findings support the idea for further preoperative genetic screening among hypertensive patients. Santulli et al. tended also to explain the relationship between G-protein-coupled receptor kinase 2 and hypertension in order to open new therapeutic and investigation spaces [16]. Regarding gene polymorphisms and response differences in antihypertensive treated patients, further investigations are recommended [17]. A more detailed discussion in behond the scope of the article, but the genetic background of cardiovascular diseases must be considered. These findings support the idea for further preoperative genetic screening among hypertensive patients.

The anesthesiologist must calculate the cardiac risk of a patient using Revised Cardiac Risk Score, age, and the type of surgery (emergency, bleeding likelihood, intraoperatively hemodynamic changes etc.), which would help to identify patients at risk and to further evaluate them.

## Preoperative cardiac evaluation

Preoperative evaluation is a crucial part of an anesthesiologist’s daily practice. This evaluation includes past medical history, current illnesses and medications, previous experience with anesthesia, previous surgery, allergies, airway evaluation, blood chemistries, and cardiac and pulmonary evaluation.

The American Society of Anesthesiologists’ Physical Status classification system categorizes patients in 5 classes depending on their physical status. The physical status varies from a healthy patient (class I) to a moribund one (class V). The American College of Cardiology and the American Heart Association have recently determined the guidelines [18] for evaluation of cardiac patients undergoing non cardiac surgery. These guidelines confirmed that active cardiac diseases include unstable or severe angina, recent myocardial infarction, decompensated heart failure, severe arrhythmias and decompensated valvular disease. According to these guidelines, urologic surgery is considered as intermediate-risk surgery with cardiac risk 1-5%. A patient without active cardiac disease having low cardiac risk needs no further evaluation, whereas the patient with active cardiac disease, poor functional capacity, intermediate-cardiac risk, and abnormal non-invasive testing can be further evaluated (radionuclide angiography, and coronaroangiography). All these examinations can be done if the patient is not having emergency surgery. If an emergency surgery is to be performed, the patient must proceed with surgery.

Another issue the anesthesiologist must deal with is chronic medications. It is established that all drugs can be continued exept angiotensine-convertase inhibitors, oral antidiabetic drugs, and antiplatelets. If the patient has coronary stents, the anesthesiologist must stop clopidogrel and continue with aspirin. Theoretically, it is reasonable to stop antiplatelet therapy 4 to 5 days before surgery in patients with an increased risk of bleeding without exposing them to the hypercoagulability. Nuttal [19] suggested that 30 days may be a minimum time interval between PCI with baremetal stents and noncardiac surgery. Rabbitts [20] concluded that surgery must be postponed after drug-eluting stent placement by more than 365 days. Warfarin must be stopped 3–5 days before surgery, substituted with low molecular weight heparine.

### Anesthetic considerations

#### *Unique urologic surgery related problems*

Major oncologic-urologic procedures are usually performed for renal, bladder, and prostate tumors, which are among the most common cancers in men [21]. Renal tumors may be diagnosed mainly in the fourth to seventh decades of life. These tumors can metastasize to bone, lungs or brain, can infiltrate nearby organs, such as liver or spleen, and can be associated with paraneoplastic syndromes. Often renal tumors invade the inferior vena cava (IVC), reaching the right atrium in about 4–10% of cases. The surgery may need cardiothoracic and vascular surgical input, particularly if the tumor is invading IVC, using cardiopulmonary bypass. The major complications of radical nephrectomy are bleeding and thrombus migration causing tumor embolism. So the anesthesiologist must place 2 large bore IVs and preferably a central line to facilitate fliud resuscitation and cardiac drugs administration. If hypovolemia occurs, decreased venous return induces decreasing end diastolic volume and stroke volume. The decreased stroke volume contributes to reduced cardiac output and decreased myocardial vascularisation, thus inducing myocardial ischemia and further decreasing contractility. Hypotension-associated tachycardia leads to shorter diastolic time and further decreases blood supply to myocardium. All these phenomena induce myocardial ischemia and decrease contractility, which can further complicate current cardiac disease. If tumor embolism is suspected (sudden decrease of ETCO2 and oxygen saturation), an intraoperative transesophageal echocardiography can be performed. During tumor preparation, often the pleura is damaged, causing open pneumothorax predisposing to postoperative respiratory complications. The anesthesiologist must be aware of this complication because a postoperative hypoxemia can further deteriorate the cardiac function in patients with poor cardiac reserves. Hypoxemia may lead to myocardial ischemia, decompensate the chronic heart failure, and induce arrhythmias. During renal cell tumor removal, the patients are often placed in lateral decubitus position. The physiological changes of this position are thought to reduce venous return and reduce preload. These changes are usually transient, but in dehydrated patients (patients suffering from heart failure under chronic diuretic therapy), patients on mechanical ventilation, and those laterally positioned (partially occluding IVC), the severity of hypovolemia is greater.

Radical cystectomy includes removal of bladder, prostate, seminal vesicles, and lymphonodes. If an ileal conduit is planned, patients are often pretreated with laxatives to clean the gut. This generally leads to dehydration, hypokalemia, and metabolic acidosis. The anesthesiologist must carefully monitor the volume status, electrolytes, and the presence of acidosis. During the Santorini’s plexus manipulation, bleeding can occur, so large bore IVs and/or central lines cannulation are preferred. The anesthesiologist must prevent both hypovolemia (which can induce low cardiac output) and volume overload (which can lead to edema, decompesation of chronic heart failure, and pulmonary edema with subsequent hypoxemia). The patient’s outcome is usually good even in patients with comorbodities [22].

The preferred surgical technique for prostate cancer removal is radical prostatectomy. This can be done by open surgery or a laparoscopic approach. Laparoscopic and robotic approaches offer less blood loss and faster recovery [23]. During open radical prostatectomy bleeding is the most important complication. It is well known that perioperative anemia can induce cardiac problems, thus a perioperative hemoglobin level around 10 g/dl is acceptable and is a gold standard. All interactions between cardiac patients and urology are summarized in Table 1.

**Table 1:** The interactions between surgical procccedure, preexsisting cardiac diseases, and the anesthesiolohgist’s role

**Clinical situations**	**Pathophysiology mechanisms**	**Clinical impact on cardiac function**	**Anesthesiologist’s role**
Bleeding hypovolemia	Hypovolemia, hypotension	New myocardial ischemia episode	Preoperative optimisation
	Tachycardia	Decompensated heart failure	Cross matched blood units
	Reduced venous return	Myocardial infarction	Large bore veins
	Decreased stroke volume	Severe arrhythmias	Central vein
	Decreased cardiac output		Invasive blood pressure monitoring
	Futher hypotension		Rapid liquids administration devices
	Anemia induced moycardial hypocontractility		Cell salvage
			Vazopressors
			Thromboelastography
Thrombus migration	Sudden increased right ventricular afterload	Deteriorate right ventricular infarction	ETCO2 monitoring
	Right infarction	Global heart failure	PEEP
	Hypoxemia		
	Arrhythmias	Cardiac arrest	Transesocardiography
			Aspiration from central catheter
Open PNX	Hypoxemia	Decompensate preexsiting cardiac and respiratory diseases	Strict comunication with surgeon
	Impaired contractility		
			Manual ventilation till pleura clossure
			Postoperative X-ray
Position (lateral decubitus)	Reduced venous return	Deteriorate cardiac function	Reduce PEEP level
			Hypovolemia correction
			Large bore veins
			Central vein
			Invasive blood pressure monitoring
Metabolic changes	Decreased cardiac output	Metabolic acidosis	Careful monitoring
	Drug induced diarrea		
	Loss of bicarbonates	Impaired cardiac contractility	Preoperative correction
	Hyper/hyponatremia	Respiratory alchalosis	Enteral/parenteral nutrition
	Hyper/hypokalemia	Arrhythmia	
	Hyper/hypochloremia		

PNX-Pneumothorax, ETCO2-EndTidalCO2, PEEP-Positive End Expiratory Pressure.

#### *Laparoscopic approach, pathophysiological effects on cardiac function*

Laparoscopic surgery [24,25] is widely used in major urologic proccedures such as radical and partial nephrectomy, living donor nephrectomy, pyeloplasty nephroureterectomy, radical prostatectomy and pelvic lymph node dissections. Advantages to this kind of surgery consist of reduced tissue trauma, decreased postoperative pain, better cosmetic results and faster recovery. During laparoscopy, carbon dioxide is insufflated allowing the use of diathermy because it is non-inflammable. During pneumoperitoneum - increased intraabdominal pressure may induce hypertension (increased afterload by vasoconstriction and released of catecholamines). The increased afterload and tachycardia are associated with increased myocardial workload, predisposing to ischemia. Further insufflations leads to reduced venous return, decreased preload, reduced end-diastolic volume. These changes, even temporary, can contribute to decrease in stroke volume and cardiac output exacerbating an existing heart failure, myocardial ischemia, and severe arrhythmias. Additional attention should be paid on positioning the patient for laparoscopic urology. During laparoscopic radical prostatectomy, the patient is placed in Trendelenburg position which can compromise ventilation and is generally associated with increased venous return, increased preload, and increased myocardial wall stress [26]. Table 2 summarizes the pathophysiological effects of laparoscopy.

**Table 2:** The pathophysiological effects of laraparoscopy on cardiac patients

**Pathophysiologic effect**	**Clinical impact**	**Patients in risk**
Tachycardia	↑ likelihood for myocardial ischemia/infarction, decompensation of arrhythmias, severe heart failure	Preexisting coronary artery disease
↑ Afterload		
↓ Preload	↓stroke volume, ↓ cardiac output, ↑ likelihood for decompensation of preexisting heart failure and coronary artery disease	Preexisting chronic heart failure
↓ Venous return		
		Preexisting chronic preoperative arrhythmia

↑: Increased, ↓: Decreased.

All these changes make cardiac patients (heart failure, coronary artery diseases, valvular disease) prone to further cardiac complications. Additional laparoscopic effects on other organs and systems are out of the article’s aim.

### Anesthesia - related problems

Patients with ischemic heart disease, severe congestive cardiac failure, and valvular insufficiency are prone to developing cardiac complications. The anesthesiologist must keep in mind the postoperative benefits and the intraoperative risks when the choice of surgical approach is discussed. The anesthetic technique must be individualized to the patient according to morbidity, mortality, current diseases, and surgical procedures.

Usually, the induction of anesthesia in patients with known coronary artery disease, chronic heart failure, and perioperative hypertension, must be gentle, avoiding tachycardia and hypotension. The sympathetic effects of laryngoscopy can be blunted using an opioid (fentanyl, sufentanil, alfentanyl), a bolus of lidocaine, and/or a β-blocker agent (esmolol). Hypotension is not preferred because it can decrease the coronary artery perfusion. Normal/ mildly elevated diastolic pressures, as well as avoiding tachycardia, are the main hemodynamic goals in patients with a known coronary occlusion. The anesthesiologist must maintain adequate hemoglobin level (10 g/dl) and optimal oxygenation. Hemoglobin levels of less than 9–10 g/dl predispose the patient to new ischemic episodes.

The impaired preoperative renal function is not a rare finding. It may be secondary to malignancy, obstruction, and dehydration. The anesthesiologist must be careful to choose the right anesthetic drugs. Pancuronium is generally contraindicated, and in patients at risk for postoperative renal failure cis- atracurium, atracurium, or vecuronium are preferred. Nephrotoxic drugs such as aminoglycosides, vancomycin, and non steroidal anti-inflammatory drugs must be avoided.

Weight loss is common in patients with urological malignancy, especially in the late phase which might have some impact on anesthetic technique. Hypoalbuminemia alters pharmacokinetics by increasing the drug’s effect [27]. Reduction of subcutaneous fat leads to decreased volume of distribution, prolonging the effect of a drug (benzodiazepines, thiopental) [28].

Major surgery for urological tumors is often associated with significant bleeding. Large bore intravenous access, central catheter, rapid infusion devices, and blood products should be available for this kind of surgery. National Institute of Clinical Excellence (NICE) has approved the use of cell salvage for radical prostatectomies and cystectomies [29]. The intraoperative coagulation status must be regularly checked, and in complex cases, using thromboelastography may be helpful in identifying the abnormality.

Intraoperative hemodynamic is probably the most important issue the anesthesiologist must deal with. There are several factors contributing to intraoperative hypotension. These factors include: preoperative dehydration (diuretics in a cardiac patient, gut preparation using laxatives, nausea and vomiting), vasodilatation and impaired cardiac output due to anesthesia, reduced preload and venous return (lateral position, mechanical ventilation, and PEEP, laparoscopy), and bleeding. The anesthesiologist must choose a hemodynamic profile calculating all these factors. Mean arterial pressure (MAP) must guarantee coronary, cerebral, and renal perfusion, but at the same time must be a factor for reduced bleeding. A rationale MAP of 60–80 mmHg usually guarantees all requirements mentioned above.

The standard ASA monitoring is used depending on patient’s morbidities. According to the patient’s cardiac conditions, the anesthesiologist may put a pulmonary artery catheter and invasive monitoring blood pressure cannulating radial artery. The indications for pulmonary artery catheter insertion are debatable in many centers. Transesophageal echocardiography is useful in evaluation of fluid responsiveness, and cardiac monitoring [30].

### Metabolic disturbances

If the patient is scheduled to undergo a radical cystectomy with an ileo-jejunal conduit, the gut must be prepared by using laxative drugs. This preparation may produce several metabolic changes including metabolic acidosis, hypernatremia, hypokalemia, and dehydration. The preexisting hypovolemia must be corrected because of severe hypotension after the anesthesia induction. Avoiding hypotension is generally advisable in patients suffering from cardiac or cerebral diseases. Hypokalemia induces premature ventricular beats as well as other arrhythmias.

After radical cystectomy with ileal-jejunal conduit, there is usually a contact of urine with gut mucosa, producing hyponatremia, hypochloremia, hyperkalemia, and metabolic acidosis.

Parenteral nutrition administration can help the patient gain weight, correcting the albumin blood level. If the gut is to be used for neobladder, enteral nutrition is not a suitable choice.

### The role of epidural anesthesia in perioperative cardiac function

Nowadays epidural anesthesia may be a suitable choice both as a supplement to general anesthesia and as postoperative acute pain treatment. Several authors [31,32] have advocated the role of epidural analgesia/anesthesia in reducing cardiac, pulmonary, and thrombotic complications. The activation of the sympathetic nervous system during surgery leads to myocardial infarction, angina pectoris, and fatal cardiac arrhythmias. Inhibition of sympathetic stimulation may reduce cardiac morbidity. Selective inhibition of the sympathetic nervous outflow to the heart can be achieved by thoracic epidural anesthesia (TEA), which blocks the segment Tl-5, and although oxygen supply to the ischemic myocardium is improved, total cerebral blood flow (CBF) is unaltered. TEA has several benefits in myocardial ischemia: anginal pain is improved; the stress response to surgery is suppressed; and the incidences of myocardial infarctions and arrhythmias may be reduced [31,32]. The mechanisms explaining benefits on myocardial ischemia are well defined [31] including decreased heart rate, reduced preload and afterload, and dilatation of coronary vessels. Hypercoagulability often occurs after major surgery and may induce perioperative cardiac dysfunction. Beneficial effects of epidural anesthesia on coagulation status have been described [33]. It is generally accepted that epidural anesthesia improves the patient’s outcome reducing perioperative complications [34].

The Cochrane meta-analysis confirmed benefits in postoperative acute pain treatment, but was less optimistic regarding other benefits [35]. This analysis showed no reduced mortality besides reduced cardiac and pulmonary complications. Use of epidural anesthesia in major urologic surgery is recommended from the authors of this article.

## Conclusion

A cardiac patient undergoing non-cardiac surgery presents great challenges to the anesthesiologists, which are analyzed in this review. The cardiac patient undergoing major urologic surgery needs a careful preoperative evaluation and optimization including volume and electrolytes correction, withholding chronic medication, and a good collaboration between the anesthesiologist and the surgeon to define the surgical procedure. The anesthesiologist must take care to evaluate intraoperative hemodynamic, risks of bleeding, patient’s position effects, metabolic disturbances, and new cardiac signs suggesting further deterioration of a preexisting cardiac disease. The role of epidural anesthesia is beneficial in this group of patients.

## Competing interests

The authors declare that they have no competing interests.

## Authors’ contributions

RD, HS, IO wrote the paper; AB, MK, AB, HL collected the literature. All authors read and approved the final manuscript.
